# Supplementary material for: An optimized protocol for isolation of high‐quality RNA through laser capture microdissection of leaf material
Source: Plant Direct. 2019 Aug 27;3(8):e00156. doi: 10.1002/pld3.156 (PMC6710646; doi:10.1002/pld3.156)
Supplement: Supplementary file 1 [file PLD3-3-e00156-s001.pdf]

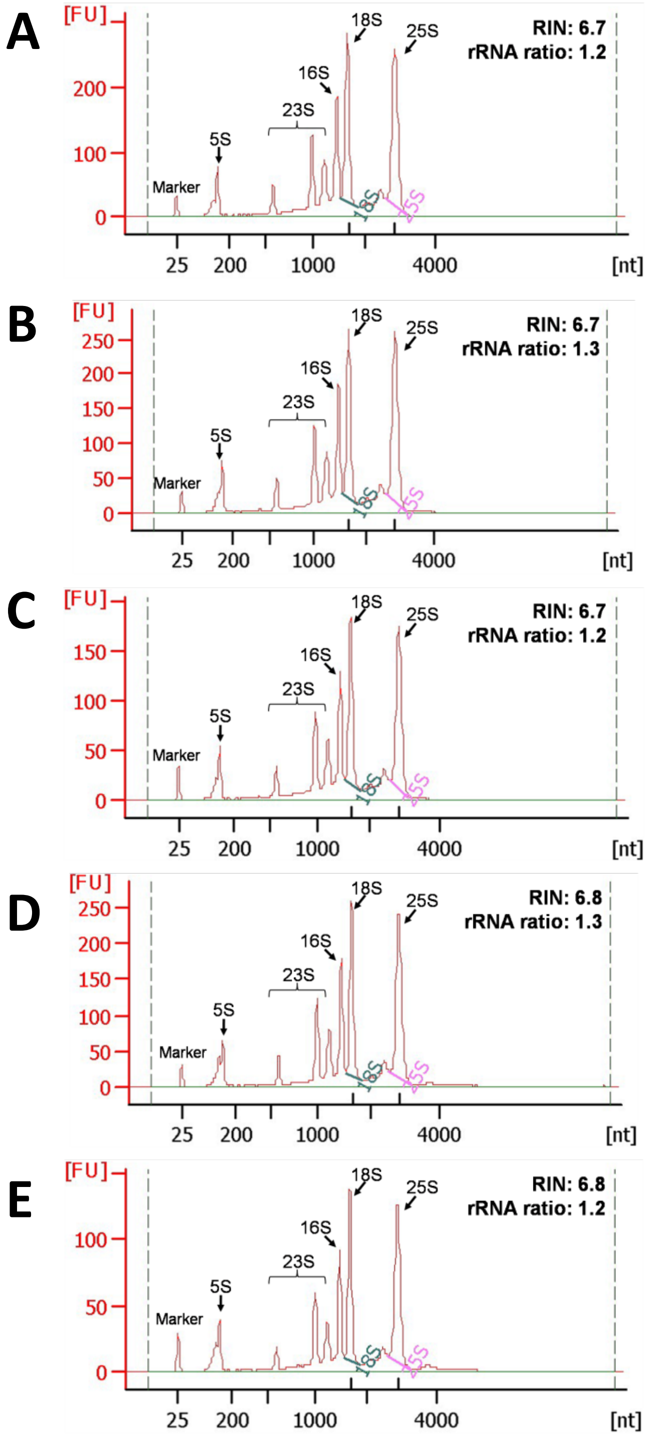

Supplemental Figure 1: RNA integrity after fixation of leaves with 100% (v/v) acetone or Farmer's fixative. Bioanalyzer traces derived from RNA extracted from snap-frozen *Arabidopsis* leaves **(A),** leaves placed in 100% (v/v) acetone for 2 **(B)** or 4 hours **(C)**, and leaves placed in Farmer's fixative for 2 **(D)** or 4 hours **(E)**. The major ribosomal RNA peaks are annotated. The y-axis shows Fluorescence Units (FU) and the x-axis nucleotide length.

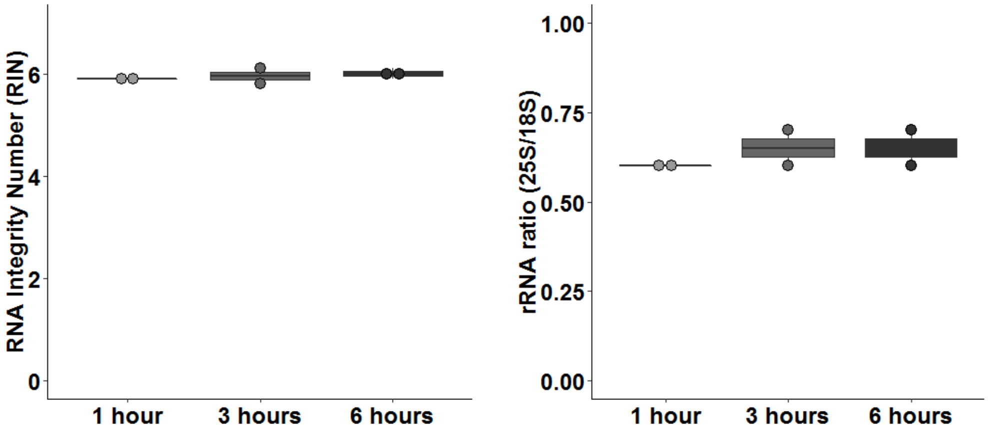

Supplemental Figure 2: Infiltration with Steedman's wax stabilizes RNA quality. RIN values (A) and rRNA ratio (B) of RNA from rice leaves after 1 hour, 3 hours, and 6 hours of infiltration in Steedman's wax at 40 ˚C.

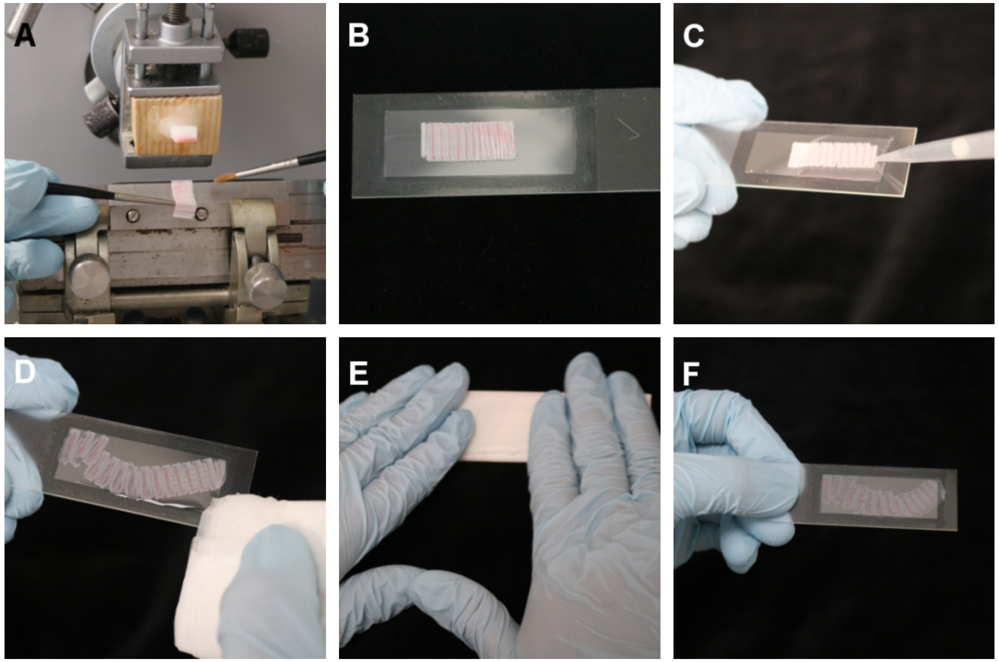

Supplemental Figure 3: Key stages in sample adhesion to slides for tissue embedded in Steedman's wax. Photographs illustrate the main steps associated with sectioning and slide preparation prior to Laser Capture Microdissection (LCM). **A.** Sectioning of Steedman's wax embedded leaf with microtome. **B.** The wax ribbon is placed on membrane slide. **C.** The wax ribbon is expanded and flattened onto the slide using DEPC-treated water at room temperature. **D.** After ribbon expansion water is removed using tissue paper. **E.** Dry sections are obtained by providing gentle pressure on each section with folded tissue paper. **F.** Exemplar slide with leaf tissue that could be used for LCM directly.

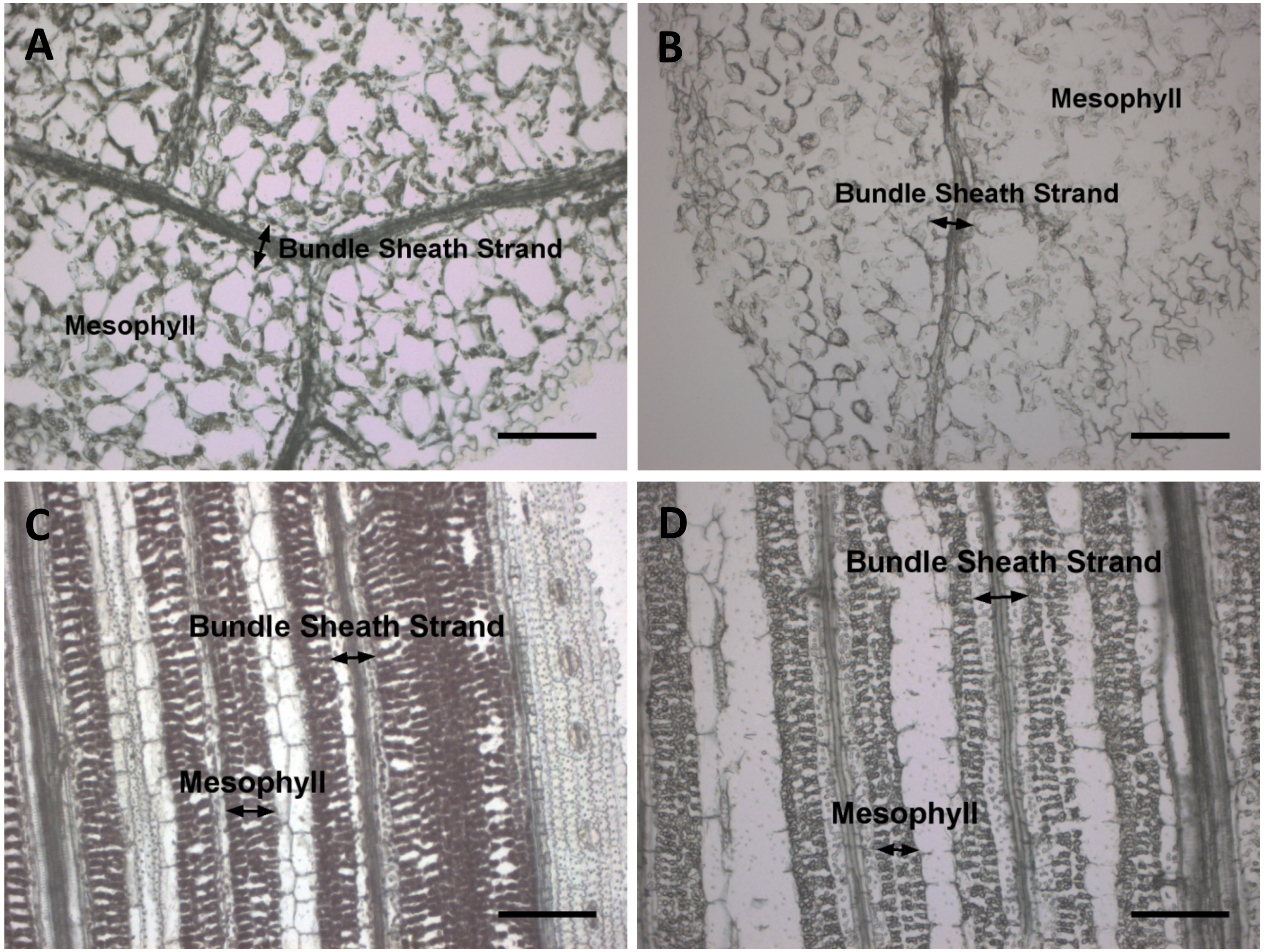

Supplemental Figure 4: Identification of BSS and M cells in Arabidopsis and rice leaf sections embedded with paraffin (A, C) or Steedman's wax (B, D). Bundle sheath strands and mesophyll cells are marked with arrows. Scale bars represent 100 μm.
